# Clinical applications and effectiveness of guided implant surgery: a critical review based on randomized controlled trials

**DOI:** 10.1186/s12903-017-0441-y

**Published:** 2017-12-13

**Authors:** Marco Colombo, Carlo Mangano, Eitan Mijiritsky, Mischa Krebs, Uli Hauschild, Thomas Fortin

**Affiliations:** 1 Private Practitioner, Milan, Italy; 2 Dental Science Department, University Vita Salute San Raphael, Milan, Italy; 3 Oral Rehabilitation Department, School of Dental Medicine, Tel-Aviv University, Tel Aviv, Israel; 4 Department of Oral Surgery and Implantology, Center for Dental, Oral and Maxillofacial Medicine (Carolinum), Johann Wolfgang Goethe-University Frankfurt am Main, Frankfurt am Main, Germany; 5 Department of Surgical and Diagnostic Sciences (D.I.S.C.) Dental School, University of Genova, Genova, Italy; 6 Oral Surgery, School of Dentistry, University of Lyon, Lyon, France; 7 Private Practitioner, Via Tasso 45, 21052 Busto Arsizio, Italy

**Keywords:** Dental implant, Oral implantology, Image-guided surgery, Computer-guided implant

## Abstract

**Background:**

Nowadays implant placement protocols are widespread among clinicians all over the world. However, available literature, only partially analyses what can be potential benefits for the clinicians and patients, often focusing just on specific aspects, such as accuracy. The purpose of this review is to compare computer guided implant placement with conventional treatment protocols.

**Methods:**

A search strategy according to the P-I-C-O format was developed and executed using an electronic MEDLINE plus manual search from 2000 up to December 2016. This review included only randomized controlled trials (RCTs) focusing on subjects treated with digital workflow for oral implant placement compared to conventional procedures. Data were extracted from eligible papers and analysed. All kinds of outcomes were considered, even patient-related and economical outcomes.

**Results:**

The search strategy revealed 16 articles; additional manual searches selected further 21 publications. Afterwards the evaluation of articles, only two studies could be selected for subsequent data extraction. The two identified RCTs analysed primary outcomes as prosthesis failure, implant failure, biological or prosthetic complications, and secondary outcomes as periimplant marginal bone loss. One RCT evaluated also the duration of treatment, post-surgical progress, additional treatment costs and patient satisfaction. The other RCT focused instead on evaluating eventual improvement of patient’s quality of life. In both selected studies, were not observed by the authors statistically significant differences between clinical cases treated with digital protocols and those treated with conventional ones. In one RCT, however post-surgical progress evaluation showed more patients’ self-reported pain and swelling in conventional group.

**Conclusions:**

Within the limitation of this review, based on only two RCTs, the only evidence was that implant survival rate and effectiveness are similar for conventional and digital implant placement procedures. This is also confirmed by many other studies with however minor scientific evidence levels. Reduction of post-operative pain, surgical time and overall costs are discussed. Authors believe that scientific research should focus more in identifying which clinical situations can get greatest benefits from implant guided surgery. This should be done with research protocols such as RCT that assess comprehensively the advantages and disadvantages of fully digital surgical protocols.

## Background

The use of the first osseointegrated implants to replace missing dental elements, almost 50 years ago, represented a huge evolution in dental rehabilitation techniques [1]. Over the years, many solutions have been proposed in order to improve the clinical performance of dental implants [2]. Implant shape has evolved with the introduction of not only cylindrical structures, of most efficient coil design and of better implant-prosthetic connections. Many surface treatments have also been suggested to modify the nanostructure of titanium, improving osseointegration processes and bone healing. The scientific literature agrees that implant-prosthetic rehabilitations have a 5-years survival rate of approximately 95% and greater than 89% after 10-years [3]. Nevertheless, the current trend in implant surgery is to further improve these clinical procedures by reducing total rehabilitation duration using, at the same time, less invasive surgical techniques. Guided implant protocols could help clinicians to simplify their procedures starting from the diagnostic phase up to the realization of the final prosthetic restoration.

The first and probably most important stage for the development of these new clinical procedures has been the introduction and the diffusion of three-dimensional (3D) imaging technique and computer technology [4]. They have allowed to improve traditional pre-surgical planning in which radiographic assessments, often through periapical and panoramic radiographs, study casts and direct inspection of the alveolar ridges, were used. The evaluation of 3D data, extracted from computerized tomography, but also more recently from optical scanner, together with modern implant planning software allow to carefully simulate surgical and prosthetic phases. Implant sites can be decided before surgery according to bone volume and quality, location of anatomical structures (nerves, vessels, sinuses), prosthetic and aesthetic evaluations [5]. Accurate one-to-one measurements of the width and height of bone in planned implant sites, as well as distances and angulations between implants from one side of an arch to another, can be predetermined without the distortions that are present in the two-dimensional radiology [6]. Implants and abutments can then be “virtually” planned, driven by knowledge of the position of the planned restoration. It also allows predetermination of prosthesis path of insertion, placement of screw chambers, componentry space, and pre-surgical abutment choices, as well as pre-surgical fabrication of individual abutments. An accurate virtual surgery planning allows sometimes to avoid bone augmentation procedures which are associated to an extension of treatment time and sometimes, unfortunately, also to major clinical complications [7]. Moreover, a careful three-dimensional positioning of the implants allows to obtain the best clinical results, especially as regards aesthetic aspects [8].

Guided implant surgery (GIS) allows to transfer planned rehabilitation project directly into surgical field. The clinician can choose between several guided methods; first, surgical guides can be divided into “static” and “dynamic”. The latter are represented by guided navigation methods in which a computer-guided navigation system helps the clinician in real time during the implant positioning through visual imaging tools on a monitor. These methods, although very interesting in future perspective are currently not particularly widespread. “Static” methods instead include the use of surgical templates that can be produced by conventional procedures, modifying a radiographic scan prosthesis, or by Computer-Aided Design/Computer-Aided Manufacturing (CAD/CAM) technologies (milling or stereolithography). Surgical guides can be tooth, bone or mucosa supported, with or without stabilization pins. Some guided systems use, for each patient, different templates with different sleeves size, while others use only one template. A further differentiation is given by the modality of implant screwing after implant site preparation: some systems provide fully guided implant insertion through the same drilling template; other methods may require the manual insertion of the implant after removing the surgical template.

Guided implants insertion often allows mini-invasive surgeries without the necessity to elevate a surgical flap. A further advantage of guided techniques is to have, at the time of surgery, a prefabricated fixed prosthesis, based on planned implants position, able to connect newly inserted implants and to easily achieve a functional and aesthetic immediate loading [9]. However even for implant guided surgery there are disadvantages which must be clearly evaluated. As first, like all new methods, this type of surgery requires a learning period for the dentist, for the technician and in general for the entire dental team. Time required for guided implant pre-surgical planning is definitely longer compared with traditional protocols. Economic aspects must also be evaluated regarding formation, instrumentation, surgical templates realization.

Due to the huge number of protocols among which the clinician can choose and their rapid evolution, the literature does not often provide accurate information about the real advantages or disadvantages that guided implant insertion protocols can provide for the clinician or for the patient. Therefore, the purpose of this review is to evaluate, through highest evidence level studies (RTCs), which are real clinical advantages and disadvantages of computer GIS compared to conventional treatment protocols.

## Methods

To ensure a systematic review of the available literature, the following steps were followed: question formulation, definition of electronic database search strategy, retrieval of publications, studies selection, data extraction and evaluation.

As a first step, the following questions, that could fulfil the purpose of this work, were formulated: which are clinical advantages and disadvantages of computer guided implant placement compared to conventional treatment protocols? Regarding medical and economic aspect, in which clinical situations guided surgery could be justified?

Based on the PICO criteria, a search strategy was developed and executed in MEDLINE (PubMed) electronic database. Search terms were selected and then grouped into categories for “Problem” – “Intervention” – “Control” – “Outcome”. The search strategy was assembled from a combination of qualified Medical Subject Headings [MeSh-Terms] as well as unspecific free-text words in simple or multiple conjunctions [Table 1]. Only works published from January 2000 up to December 2016 in English language were considered. An additional manual search of the bibliographies of all selected full-text articles was performed. Furthermore, searching was also conducted in dental literature using free-text terms and phrases.

**Table 1:** Overview of the electronic search strategy including source of database, timeline, and P-I-C-O definition for study selection

Database	MEDLINE (PubMed)
Timeline	From January 2000, up to December 2016
P-I-C-O	Problem {(« Dental Implant » [MeSH]) OR (« Oral Implant » [MeSH]) OR (« endosseous implant » [MeSH]) OR (« implant placement ») OR (« dental implant graft » [MeSH]) OR (« dental implant graftless ») OR (« dental implant flapless » [MeSH]) OR (« dental implant flap » [MeSH]) OR (« Alveolar Ridge Augmentation/methods » [MeSH]) OR (« Alveolar Bone Loss/surgery » [MeSH])} AND Intervention {(« Image-guided surgery » [MeSH]) OR (« computer assisted surgery» [MeSH]) OR (« guided surgery ») OR (« computer guided ») OR (« surgical innovation ») OR (« Computer-Aided Design »)} AND Control {(« Conventionnal surgery ») OR (« open flap ») OR (“muccoperiosteal flap”) AND Outcomes {(« accuracy » [MeSH]) OR (« patient-based outcome ») OR (« costs, costs analysis » [MeSH]) OR (« treatment duration ») OR (« treatment ease ») OR (« practitioner-based outcomes ») OR (« success » [MeSH]) OR (« survival » [MeSH]) OR (« postoperative sequel ») OR (« aesthetics, dental » [MeSH]) OR (« prosthetic outcome »)

Only randomized controlled studies comparing digital workflow for oral implant placement with conventional procedures in alveolar crest were included. Inclusion criteria were also:

- at least five patients in each group;
- minimum follow-up of 6 months after loading;
- presence of clinical, radiographic, patient-centred outcomes or economical evaluations;

Studies that dealt with zygomatic or pterygoid implants, orthodontic mini-implants were outside the scope of this review.

All authors independently read and exanimated titles and abstracts of all studies identified with electronic and hand search and recommended their inclusion or exclusion according to defined criteria. When at least one author considered that a publication met inclusion criteria, full text was obtained and evaluated for its eligibility. Disagreements about inclusion or exclusion were resolved by discussion. Afterwards It was also conducted a search within references of full-text publications evaluated. After assessment process, the following data were extracted from eligible studies: authors, year of publication, study design, patients’ population, type of surgical intervention, primary and secondary outcomes. All the authors performed full-text articles evaluation and data extraction together during 1st Conference meeting of the Digital Dental Society (DDS) in Milan, Italy, 16th–17th September 2016; all disagreements were resolved by discussion. In the same way, it has been assessed the risk of bias for eligible studies using Cochrane Collaboration’s tool for assessing risk of bias [10].

## Results

### Included studies

**Fig. 1:**
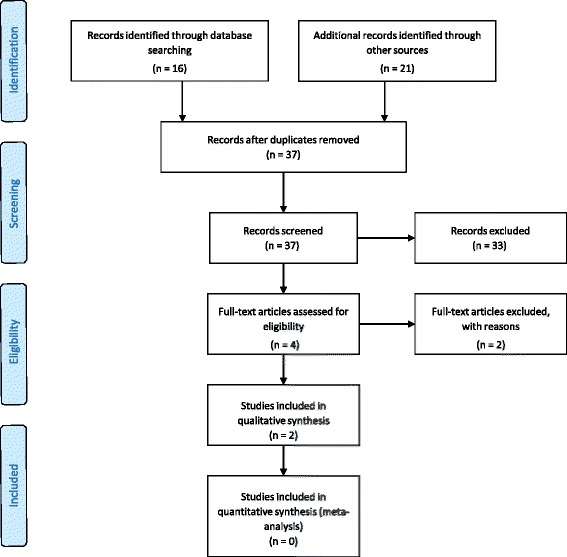
Flow-chart depicting the electronic and manual search result

Evaluation process of articles selected for this review is schematically shown in Fig. 1. The search strategy carried out in MEDLINE (PubMed) electronic database revealed 14 articles; additional manual searches selected further 21 publications. Afterwards the evaluation of the 37 abstracts there were found only four works who respected inclusion criteria. Of these publications, after full text analyses, two were excluded as one did not present a follow-up period [11], while the other did not show a control group in which conventional surgical procedures were used [12]. Finally, only two papers complied with all inclusion criteria [13, 14]. Data extracted from these two papers were reported in Table 2.

**Table 2:** Summary table of included studiesᅟ

Study	Study design	Population	Intervention	Surgical guide	Primary outcomes	Secondary outcomes
Vercruyssen et al. 2014 [13]	RCT, 1y F/U	59 patients (72 fully edentulous jaws)	60 jaws divided in 5 groups (each one *n* = 12), treated with 5 different surgical template, 4–6 implants for each jaw	2 groups bone-supported stereolithographic templates, 2 groups mucosa-supported templates, 1 group pilot-drill template	Prosthesis failure, implant failure, biological or prosthetic complications	Peri-implant marginal bone loss, clinical implant evaluation (BOP, paque, pockets), patient’s quality of life improvement
Pozzi et al. 2014 [14]	RCT, multicentrical, 1y F/U	51 patients, fully or partially edentulous jaws (some post-extractive placement)	25 patients treated with guided implant surgery	Stereolithographic surgical templates, stabilized with silicon index and 2–3 anchor pins	Prosthesis failure, implant failure, biological or prosthetic complications	Peri-implant marginal bone loss, time and sessions for rehabilitation delivery, complication time, post-surgical pain, swelling, consumption of painkillers and patient satisfaction, additional treatment cost

### Descriptive analysis

Two RCTs could be selected for analysis [13, 14]. They both compare implant placement with 3D planning and dedicated digital guide versus free-hand placement (conventional placement). They both evaluated success rate (prosthesis failure, implant failure, biological or prosthetic complications) with different study design.

Vercruyssen et al. [13] performed a randomised 6-arm, non-blinded, controlled trial with 59 fully edentulous patients for the placement of 4 to 6 implants in the lower or upper jaw. Evaluation was done 1 year after placement of the final restoration. There were two arms with bone-supported guide (24 patients) of two different brands, two arms with mucosa-supported guide (24 patients) of two different brands, one arm with free-hand navigation (12 patients) and one arm with guides only for pilot drill (12 patients). In the free-hand group implants were inserted using only images and measures from the planning software as a reference. In both mucosa groups, flapless surgeries were performed. The success rate at 1 year from the final loading was similar in all groups with no implant lost and then no significant difference. Evaluation of bleeding and pocket probing depth are also recorded after 1 year of follow-up but without any statistically significant difference between groups. No significant differences in peri-implant marginal bone loss could be also observed. For all treatment groups, a significant improvement in quality of life was observed at 1-year follow-up but no differences between groups were observed. This study presents a high degree of risk of bias only about randomization process especially as regard the assessment of the primary and secondary outcomes through the follow-up period.

Pozzi et al. [14] performed a randomised 2-arm, non-blinded, controlled trial with 51 fully or partially edentulous patients. Twenty-six patients were treated with conventional protocol, 25 with guided techniques. In both groups, whenever possible, flapless surgeries were performed. However, in some cases, depending on the operator’s judgment, flaps had to be elevated to better check implant sites. This occurred mostly in the control group where the implants were inserted free-hand. Patients were treated by 3 practitioners (20 and 20 patients for two clinicians and 11 for the last one) achieving a multicenter study design. For data analysis patients’ treatment were categorized, depending on the different clinical conditions, in different degrees of complexity (simple cases had almost 9 mm bone height and 7 mm bone width, complex cases had bone height between 7 and 9 mm and less than 7 mm of bone width). It must be noticed than in the computer-guided group more post-extractive implants, more complex cases and full edentulous maxillae were treated compared with conventional surgery group. One year after definitive prosthetic placement, authors didn’t find statistically significant differences between groups for the number of patients who had implant failures (1/26 in conventional versus 0/25 in computer-guided), for the number of patients experiencing complications (4/26 versus 5/25) and for peri-implant bone loss (0.80 +/− 0.29 mm for the conventional group versus 0.71 +/− 0.25 mm for guided). The treatment overall duration in days (from CBCT to provisional prosthesis delivery) was similar for the two groups. Statistically significant more pain and swelling was reported in the conventional group (*P* = 0.002 for postoperative pain, *P* = 0.024 for postoperative swelling). Surgical time from anaesthesia to suture was similar in both groups but we have to keep in mind that more complex cases were treated with guided surgery. Satisfaction, aesthetic and function, of the patient 1 year after prosthetic placement were the same for the two groups. For the computer-guided group, there were extra costs due to the protocol. Bias sources in this work originate mainly from the non-blinded outcomes evaluation and from the matter that, despite patients’ randomization, the computer-guided surgery group has more cases considered complex compared to the control group.

## Discussion

As regards the accuracy of the digital guided implant surgery, several works have been published in recent years with the aim to scientifically assess the accurateness of these techniques. Cassetta and his colleagues published two works evaluating the accuracy of a computer-designed surgical guide comparing three-dimensional positions of planned and placed implants. In the first one they compared preoperative and postoperative computed tomographic images of 116 implants [15]. They observed quite high deviation values between the postoperative position and the preoperative plan at the coronal and apical portions of implants, as well as in the angulation of implants. Even if these deviations do not seem to have clinical significance authors concluded suggesting the necessity of always keeping a safety zone of at least 2 mm to avoid critical anatomical structures injuries. In the other study they assessed, in 28 completely edentulous subjects, the influence of some clinical factors in determining the precision of the surgical guide and of implants inserted, through the comparison of pre-operative and post-operative computed tomography (CT) [16]. The effect of surgical management of the guide (fixed or unfixed), arch (maxilla or mandible), and smoking habit (normal or hyperplastic mucosa) on accuracy was evaluated. They observed that in maxilla, thanks to greater supporting surface, and with the fixation of the surgical guide, accuracy of the guides was improved. They also reported a greater global coronal and global apical deviation in smoking patients due to the increased thickness of mucosa. Another work tried to evaluate the accuracy of computer-generated and conventional surgical guides using a randomized split-mouth design [17]. Each of the ten patients in this study were randomly selected for CAD/CAM-guided implant placement on their right or left side of the mouth. Conventional guides were used on the contralateral side. The authors concluded that implants placement using CAD/CAM surgical guides provided greater accuracy in a lateral direction than conventional guides. In addition, CAD/CAM guides showed less variability of deviation values, from implant planned locations, than conventional guides. Regarding implant optical navigation systems accuracy, in literature there are only few publications. A pilot study of Wittwer has tested one of these systems in 20 patients [18]. For each patient four implants were inserted in the intraforaminal region using a flapless optically guided procedure. Evaluating the CT of the patients before and after surgery, it was assessed the accuracy of the described system. The results suggest that this type of surgical procedure can be a viable and safe alternative in clinical cases with adequate amount of bone. However, in cases where there are irregularities in bone structures it can be less predictable.

Schneider and his group in 2009 published a systematic review of the literature available at time considering eight articles regarding accuracy [19]. All studies included in this review indicated a reasonable mean accuracy with however relatively high maximum deviations. This variability seemed to depend mainly by guided surgical technique chosen and especially by type of template stabilization. CAD/CAM surgical guides showed better degree of accuracy than conventional guides. Recent literature reviews have confirmed these observations regarding the precision of the various computer-guided surgery systems [20, 21]. These revisions have analysed 19 and 24 publications respectively involving different static image guidance systems, finding similar results. Meta-analysis of the accuracy revealed a mean error of about 1 mm at the entry point and of about 1.3 mm at the apex. However, even in these works, maximum deviation values are relevant. Significant differences for all deviation parameters were found for the number of templates used in favour of single template protocols. Less deviation values were also observed when more fixation pins were used.

From the data in the literature we can state that the computer-GIS has good accuracy levels, however because of the still significant deviations it is crucial the choice of the most appropriate surgical-guided protocol and its scrupulous execution. This allows on the one hand to avoid serious complications such as penetrating nerves or critical vases, on the other to be able to apply these protocols even in complex cases such as those with severe bone atrophy.

Computer-GIS is often associated with flapless implant insertion. Although in the literature there are no long-term studies that compare directly the success rate of conventional and flapless implant placement, many works seem to agree that the implant survival rates are comparable regardless of the type of implant protocol chosen. The two articles included in this review, despite the short period of follow-up, have highlighted a lack of statistically significant difference between the two clinical procedures about to the success rate of implant-prosthetic rehabilitations [13, 14]. Further confirmations of these observations were provided by Berdougo and co-workers in a study of 2010, which, evaluated retrospectively 552 implants placed in 169 patients. They founded no statistically significant differences in the cumulative survival rate after 1 to 4 years of follow-up [22] between implants inserted with flapless guided systems versus conventional open-flap implant surgery. Same conclusions were reached by a systematic review published in 2012 in which were included 28 studies on computer-guided implant placement with a total of 4032 analysed implants [23]. This systematic review indicated that guided placement had at least as good implant survival as conventional protocols showing also a significantly decrease pain and discomfort in the immediate postoperative period, but probably due to the use of flapless procedures themselves. In this review however, it was also suggested that this technique still requires a good preparation of operators to reduce as much as possible unexpected procedure-linked adverse events during guided implant placement.

Thanks to implants planning and placement in accordance with the prosthetic treatment plan, GIS may bring significant benefits to prosthetic rehabilitation procedures. Provisional prosthesis can be prepared before clinical phases so that, immediately after surgery, functional loading of the newly inserted implants can be easily achieved. It is also possible to use a single implant abutment both for the provisional and for the definitive rehabilitation, allowing time and costs reduction, but above all improving clinical outcomes especially in the esthetic zone [24, 25]. Many articles highlight how potential prosthetic benefits are greater, especially in the case of fully edentulous and immediate loading patients’ rehabilitations. In a paper published in 2007, Sanna described an immediately loaded CAD/CAM protocol on 30 consecutive patients, evaluating cumulative survival rate and marginal bone remodelling after 5 years [26]. Results seem to suggest that proposed treatment protocol allows a good survival rate of implant supported rehabilitations in fully edentulous patients. In another study of van Steenberghe and collaborators 27 patients with fully edentulous maxillae were recruited and treated with a digital guided protocol [27]. It was possible to transfer, with good accuracy, implant treatment planning to the surgical field allowing functional loading of implants immediately after their application. After a year, all implants and overlying prosthesis were considered successful.

However, prosthetic guided surgery advantages are today still largely theoretical and mostly linked to the opinion of some authors and not deriving from articles with high scientific evidence such as systematic reviews. In addition, many of these works highlight the important number of complications, especially with prostheses, that relieve these protocols [28]. Tahmaseb, in his review, reported intraoperative or prosthetic complications in 36.4% of the treated cases [21]. These reported complications included surgical complications like guide fractures or prosthetic complications like misfits with frameworks and prosthetic fractures. The rate of complications seemed to be closely related to the surgical technique learning curve.

The use of dedicated implant planning software and of guided surgery could sometimes easily avoid bone augmentation procedures. Many authors have published several works in which it was possible to insert implants with guided surgical protocols in atrophic areas. Fortin reported 98% implant survival rate after 4 years in partially edentulous cases with severely resorbed posterior maxilla avoiding sinus augmentation procedure [7]. Implants have been inserted with a CAD/CAM surgical template, based on digital planning, exploiting anterior or posterior wall or the septa of the sinus as well as the palatal curvature. During the 4-year observation period, no complications were recorded, no implants were lost, and there was no infection or inflammation.

In order to evaluate the benefits that guided surgery could provide they have to be assessed costs that these procedures imply. It must be considered an initial investment to buy the technology, but also the cost and the time for clinical team training. Finally, there will be a cost for digital workflow for each clinical case. We consider important that the clinician should be well prepared with regards to both new digital but also conventional procedures because they may need to be applied in case of any unforeseen event during guided surgery procedures. Even if the duration of the surgical intervention may be shorter with guided surgery compared to conventional techniques, it seems that much more time must be invested in the preoperative planning. If guided surgery can avoid bone grafting procedures, it can reduce the overall treatment cost. A significant treatment time reduction could as well reduce the overall costs and compensate some of the additional costs. Depending on the workflow an immediate reconstruction might as well lead to a reduced time amount, which is necessary for the completion of the final reconstruction. Unfortunately, in the literature cost-effectiveness report are not present also due to the multitude and diversity of the different protocols proposed.

Despite enthusiastic expectations are often associated with computer aided implant placement, this review revealed an important lack of high level scientific studies that could compare conventional implant protocols with digital workflows. Some RCTs investigations were retrieved but only two with at least 6 months of follow-up after implant placement could be included in this review [13, 14]. Most of minor evidence level studies are focussed on case series, technical notes or specific aspects of computer-GIS often without any comparison with conventional protocols. Furthermore, many clinical trials show results, but do not go to investigate how different protocol variables compete determining them. We believe that many factors are responsible for determining the effectiveness of GIS, from diagnostic and planning phases to surgical intervention. Every aspect should be analyzed more carefully to scientifically assess which GIS protocol could provide best performances in the specific clinical situation.

The only evidence that was retrieved from the only two papers considered eligible for our review, is that clinical outcomes are similar for the image-guided implant placement and for the conventional procedure after a follow-up period of at least 6 months. Reduction of post-operative pain and surgical time are discussed. For Pozzi and co-workers, more pain was reported, 3 days after surgery, in the open flap control group despite of in flapless/miniflap computer-guided group more post-extractive implants, more complex cases and full edentulous maxillae were treated [14]. Vercruyssen and co-workers reported no significant differences in patient satisfaction, but this appears to be linked only with the questionnaire that is fulfil 1 year after restoration placement [13]. Another work published in 2010 by Arisan et al., regarding patient’s related outcomes, found similar evidences [11]. They showed statistically significant differences in favour of the group of patients treated with flapless guided surgery compared to those treated with conventional open-flap procedures, regarding the number of analgesics consumed, post-operative pain and haemorrhages. Also, Fortin and collaborators, in a controlled trial founded a significant difference in pain measurements, with higher scores on questionnaires in open-flap surgery group comparing with flapless guided surgery group [29]. In contrast, an RCTs published in 2010 show worse results in guided implant flapless groups in comparison to conventional open flap implant surgery [12].

## Conclusions

Based on the results of the screened literature it is evident that the overall scientific evidence in the field of image guided implant placement is low. Only two RCTs with at least 6 months of follow-up could be identified. The only evidence that is retrieved is there are no statistically significant differences between conventional and computer-guided implant placement procedures, both for patient outcomes and implant survival rate. Reduction of post-operative pain and surgical time are discussed. Consequently, certain clinical recommendations cannot be given. However, indications for guided-implant surgery could be the need for minimally traumatic or flapless surgery, optimal implant positioning and immediate loading.

Further research should be designed as RCTs avoiding any possible sources of bias. These studies should take into consideration not only rehabilitation success and potential complications, but also patient-reported outcome measures (PROMs), and economic aspects. The purpose of these studies should therefore be to clarify clinical indications of guided implant surgery.

## Abbreviations

3D: Three-dimensional
CAD/CAM: Computer-Aided Design/Computer-Aided Manufacturing
CT: Computed tomography
GIS: Guided implant surgery
RCT: Randomized Controlled Trial
